# Resident physician duty hours, resting times and European Working Time Directive compliance in Spain: a cross-sectional study

**DOI:** 10.1186/s12960-023-00857-x

**Published:** 2023-08-24

**Authors:** D. A. Sanchez Martinez, J. P. Carrasco Picazo, P. D. Estrella Porter, R. Ruiz-Montero, A. H. Aginagalde Llorente, E. García-Camacho, J. Navarro, A. Cerame del Campo

**Affiliations:** 1 Consejo General Colegios Oficiales de Médicos de España, Madrid, Spain; 2 Department of Medical Oncology, IMIB-Arrixaca, Murcia, Región de Murcia Spain; 3 Department of Psychiatry, Hospital Clínico Universitario de Valencia, Valencia, Spain; 4 Department of Preventive Medicine, Hospital Clínico Universitario de Valencia, Valencia, Spain; 5 Department of Medical and Surgical Sciences, University of Cordoba, Cordoba, Spain; 6 Maimonides Biomedical Research Institute of Cordoba (IMIBIC), Cordoba, Spain; 7 Ministry of Health of the Basque Government, Sub Directorate for Public Health and Addictions of Gipuzkoa, San Sebastian, Spain; 8 Department of Cardiology, Complejo Hospitalario Universitario Toledo, Toledo, Spain; 9 Intensive Care Department, Hospital Universitario Virgen del Rocío, Seville, Spain; 10 Plan de Atención Integral al Profesional Sanitario Enfermo, Servicio Madrileño de Salud, Madrid, Spain

**Keywords:** Internship and residency, Shift work schedule, Legislation, Spain, European Working Time Directive

## Abstract

**Background:**

There is a growing interest in understanding the impact of duty hours and resting times on training outcomes and the well-being of resident physicians. However, to this date no state-wide analysis exists in any European country.

**Objectives:**

Our aim is to describe the shift work scheduling and to detail the degree of compliance with the Spanish legislation and the European Working Time Directive (EWTD) of Spanish resident physicians, focusing on territorial and specialty distribution.

**Material and methods:**

A descriptive cross-sectional analytical study was designed through an online survey adapted from the existing literature.

**Results:**

Out of the 2035 surveyed resident physicians undergoing PGT in Spain, 80.49% exceeded the 48 h per week limit set by the EWTD and 13% of them did not rest after a 24-h on-call shift. The mean number of on-call shifts in the last 3 months was 15.03, with the highest mean reported in Asturias, La Rioja, and Extremadura. 51.6% of respondents had a day-off after a Saturday on-call shift. Significant differences are observed by region and type of specialty.

**Conclusion:**

Resident physicians in Spain greatly exceed the established 48 h/week EWTD limit. Likewise, non-compliance with labor regulations regarding mandatory rest after on-call duty and minimum weekly rest periods are observed.

**Supplementary Information:**

The online version contains supplementary material available at 10.1186/s12960-023-00857-x.

## Practice points

- This study is the first to describe and analyze working conditions of resident physicians in Spain and to portray the degree of compliance of this country’s postgraduate training system with European working regulations.
- The vast majority of resident physicians (80%) exceed the maximum number of working hours set by national and European legislation.
- A significant percentage (13%) of resident physicians cannot rest after a 24-h shift working at least 32 h non-stop, a higher value is found in surgical specialties.
- Half of respondents were not able to enjoy mandatory weekly breaks.
- The possible consequences for both the well-being of residents and the quality of training are discussed.

## Introduction

The Spanish Postgraduate Training (PGT) system, known as MIR system, dates back to the 1970s and is administered by the Spanish Ministry of Health. Unlike other European countries, it does not depend on Higher Education Institutions. The system offers around 8000 PGT positions per year and access is granted through a national competitive exam which takes place once a year. Resident physicians are considered workers with special training contracts [1] and, therefore, they are subject to a labor regulation where the objective of their contract is to acquire the necessary competencies and skills to become specialists. Training takes place in the Spanish National Health Service hospitals and out-patient clinics and follows the guidelines from the National Specialty Boards. Training lasts from 4 to 5 years depending on the specialty.

Resident’s duty hours in Spain comprise a regular working schedule of 37.5 h per week and mandatory on-call shifts [1]. The number of mandatory on-call shifts is regulated by each medical specialty’s PGT program, but the usual number ranges from four to six 24-h on-call shifts. The most recent duty hours regulations in Spain were transposed from the European Working Time Directive [11] into a national law for doctors and specialists-in-training in 2008. According to Spanish Law [2], doctors cannot work for more than 48 h per week and need to have resting times per day (at least 12 h), per week (at least 36 h) as well as annual leave (at least a month).

However, there is a discrepancy between the aforementioned regulations: the number of mandatory 24-h shifts dictated by PGT programs (4–6) is equivalent to working 50–70 h per week, which would be in breach of both the European and Spanish regulations and which, in turn, contravenes the primacy principle of EU and national law. Moreover, this situation takes place in a context where there is not a duty-hours oversight system on a national, regional, or local level despite the fact that it is contemplated in said legislation. To this date there is no publicly available data source or study describing the number of residents’ working hours.

Given the importance of the possible consequences of overtime [4, 7, 8] and the lack of knowledge about the situation in Spain, the present study has the following objectives. Firstly, to describe the number of shifts performed by resident physicians in Spain and to problematize the negative impact which this number of hours entails. Secondly, to describe compliance with the daily and weekly breaks taken by resident physicians compared to those set in national and European law. Finally, to analyze the difference by demographic variables, specialty and region, in both the number of on-call duty shifts and compliance with breaks.

## Material and methods

A descriptive, observational and cross-sectional study was carried out through an online self-administered survey (Additional file 1). The target population were Spanish resident physicians undergoing PGT who started their specialist training during the years 2017–2021 and who had to do mandatory 24-h on-call shifts in accordance with their PGT program.

*Inclusion criteria* Resident physicians undergoing PGT during the study period in Spain. Exclusion criteria: resident physicians undergoing PGT in specialties which do not have 24-h on-call duty in Spain.

The reference population was obtained through the Spanish Health Ministry Database of PGT positions allocation [15] for the years 2017 to 2021, both years included. It was confirmed that there were no possible duplicates due to abandonment. For the 2017 cohort, only 5 years-of-duration specialties were considered.

Additional file 2 contains a table with the list of specialties, number of resident physicians practicing at the time of the survey, number of responses and % of responses. Additional file 3 contains a table with the list of Spanish regions, known as autonomous communities (AC), number of resident physicians practicing at the time of the survey, number of responses and the percentage of responses. The following specialties can be chosen by medical doctors or by other professions so the number of places which are shown are not solely covered by physicians: Clinical Analysis, Clinical Biochemistry, Immunology and Microbiology and Parasitology.

The study was conducted between the 1st and 31st May 2022.

A non-probabilistic quota sampling was used. A set of milestones to be achieved by the survey respondents was established for the selection of a representative sample with key variables: specialty and region. The objective was to saturate the established quota (specialty or region), which may have been expanded during the study according to the pre-set milestones and the degree of participation. A minimum of 5% of respondents was expected in each of the quotas.

For the sample size calculation, 1014 subjects were randomly selected to estimate a 95% confidence and a precision of ± 3 percent units, a population percentage considered to be around 50%. It has been anticipated to have a replacement rate of 0%.

A survey was adapted from available national literature [5, 6] and was revised by a group of experts from the Spanish Medical Organization (CGCOM) and piloted by 30 subjects. The final survey was hosted on a website of said entity, which required a two-layer secure identification system which ensured the respondents identity and registration for practice in order to access the survey. Only one survey could be completed per respondent. A response rate of 5% was estimated.

The survey was disseminated through the Spanish regional medical councils to all active resident physicians by mail as well as through informal communication channels, like instant messaging and other apps. A first mass mailing was carried out on the 1st May 2022 and two reminders were made two and three weeks later.

A descriptive analysis of the variables was carried out, indicating absolute and relative frequencies for qualitative variables and relative frequencies for qualitative variables, as well as arithmetic mean and standard deviation (SD) for quantitative variables and percentage and standard deviation (SD) for qualitative variables. For the bivariate analysis, the Chi-square test was used to compare qualitative variables and the *t*-Student test for quantitative variables.

The dependent variables were dichotomized (12 or less 24-h on-call shifts in the last 3 months, mandatory rest in the past 5 on-call shifts, day-off after a Saturday 24-h on-call shift and not working after an on-call shift) and their strength of association with the independent variables (type of specialties, gender and cohort of PGT access) was estimated in OR through logistic regression. The strength of association between the dependent and independent variables was estimated in OR through logistic regression. All hypothesis contrasts were bilateral and those contrasts with a *p* < 0.05 were considered significant. The data were processed with the statistical program Stata 17 y R 4.2.

Participants consented to the use of their data in an anonymous and aggregated form before taking part in the study. The study was authorized by the Spanish Medical Organization’s General Assembly which is the highest ethical and deontological body of physicians in Spain (Consejo General de Colegios Oficiales de Médicos de España) for its implementation and dissemination to members who were eligible to respond to the survey.

## Results

From a calculated universe of 30,377 doctors undergoing PGT in Spain from 2017 to 2021, 2035 responses were obtained, of which 65.36% identified as females. According to their year of start of residency the responses were, 85 out of 1468 in 2017, 316 out of 6513 in 2018, 444 out of 6794 in 2019, 604 out of 7615 in 2020, and 586 out of 7987 in 2021 (Additional file 2).

**Fig. 1:**
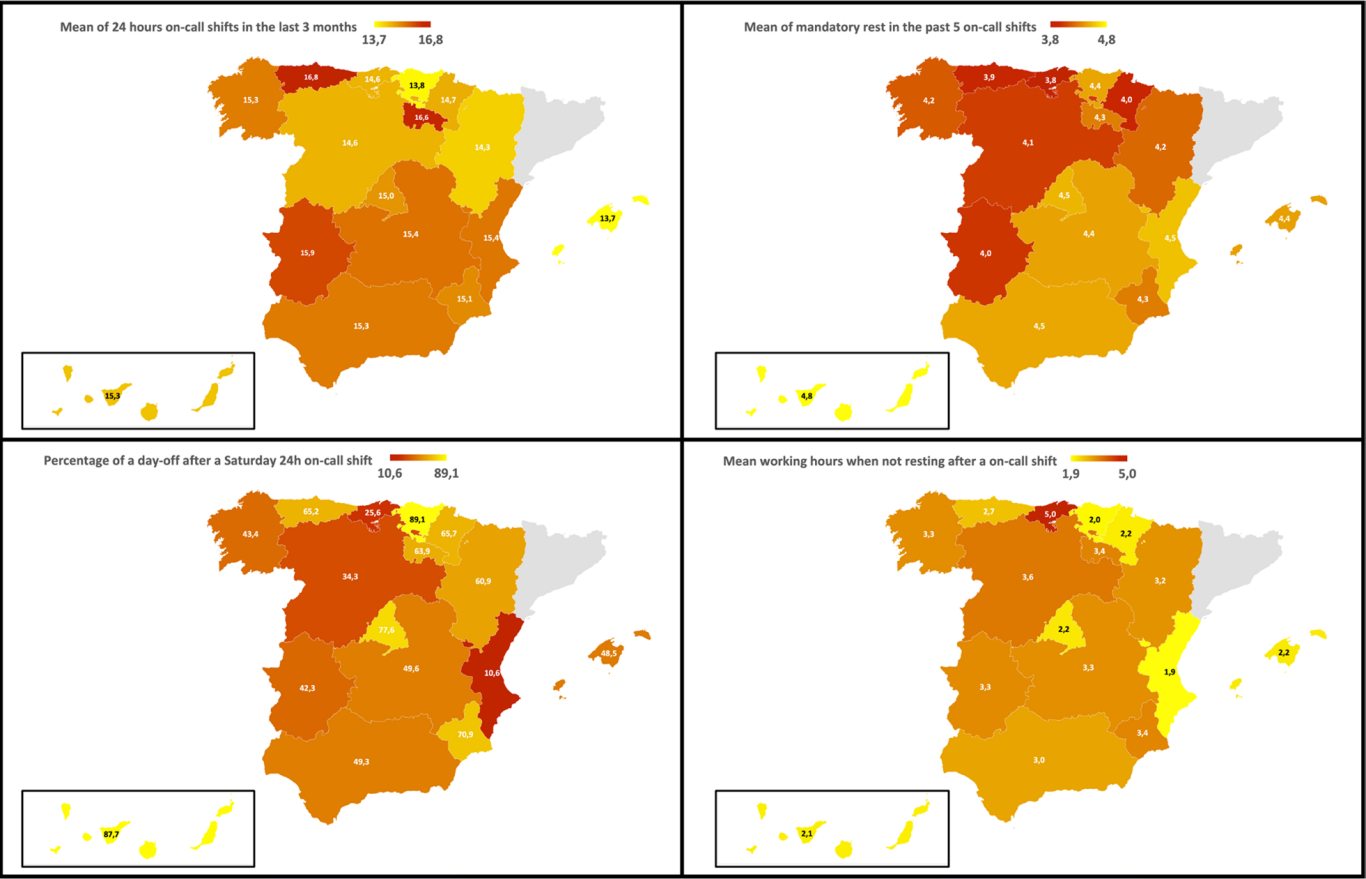
Visual summary of the relevant indicators surveyed per region

The mean number of on-call shifts made by trainees during the last 3 months was 15.03 on-call shifts. This mean was highest in men 15.26 (CI95% 15.02–15.50), and in the cohort of 2017 15.46 (CI95% 14.67–16.25). By regions, the highest mean of on-call shifts in the last 3 months was reported in Asturias 16.83 (CI95% 16.19–17.47), La Rioja 16.62 (CI95% 16.16–17.08) and Extremadura 15.94 (CI95% 15.38–16.50) (Table 2). By group of specialties the highest mean number corresponds to Surgical 16.61 (CI95% 16.19–17.03), Surgical-Medical 15.25 (CI95% 14.71–15.79) and Other Clinical specialties 15.06 (IC95% 14.85–15.27) (Tables 1, 2 and Fig. 1).

**Table 1:** Variables per speciality

Variables	Total number of responses	Mean of 24-h on-call shifts in the last 3 months (CI 95%)	*p*	Mean of mandatory rest in the past 5 on-call shifts (CI 95%)	*p*	Percentage of a day-off after a Saturday 24-h on-call shift (CI 95%)	*p*	Mean working hours after an on-call shift (CI95%)	*p*
*Type of specialties*									
Primary care	663	14.49 (14.27–14.71)	–	4.66 (4.60–7.72)	–	63.91 (60.08–67.58)	–	2.41 (2.07–2.75)	–
Other clinical	937	15.06 (14.85–15.27)	< 0.001	4.55 (4.48–4.62)	0.089	56.20 (52.92–59.42)	0.002	2.29 (2.04–2.54)	0.57
Surgical	218	16.61 (16.19–17.03)	< 0.001	3.26 (3.01–3.51)	< 0.001	22.64 (17.31–28.98)	< 0.001	5.31 (4.76–5.86)	< 0.001
Surgical-medical	159	15.25 (14.71–15.79)	0.008	3.26 (2.94–3.58)	< 0.001	26.85 (20.08–34.83)	< 0.001	4.45 (3.84–5.06)	< 0.001
Diagnostic/ laboratory	58	14.22 (13.13–15.31)	0.54	4.41 (4.08–4.74)	0.14	52.94 (38.60–66.84)	0.12	2.21 (1.33–3.09)	0.72
*Gender*									
Female	1330	14.91 (14.74–15.08)	–	4.39 (4.32–4.46)	–	55.29 (52.54–58.01)	–	2.73 (2.51–2.95)	–
Male	705	15.26 (15.02–15.50)	0.022	4.25 (4.15–4.35)	0.017	48.24 (44.44–52.06)	0.003	2.98 (2.66–3.30)	0.20
*Cohort of PGT access*									
2017	85	15.46 (14.67–16.25)	0.13	3.95 (3.60–4.30)	0.001	39.76 (29.36–51.12)	0.022	3.62 (2.76–4.48)	0.067
2018	316	14.96 (14.57–15.35)	0.73	4.30 (41.15–4.45)	0.10	50.17 (44.39–55.94)	0.38	3.00 (2.55–3.45)	0.36
2019	444	15.10 (14.77–15.43)	0.28	4.24 (4.11–4.37)	0.011	50.80 (46.01–55.59)	0.43	2.94 (2.5–3.33)	0.42
2020	604	15.10 (14.89–15.31)	0.25	4.40 (4.30–4.50)	0.51	57.19 (53.08–61.2)	0.18	2.61 (2.26–2.96)	0.64
2021	586	14.88 (14.61–15.15)	–	4.45 (4.36–4.54)	–	53.30 (49.13–57.42)	–	2.73 (2.39–3.07)	–
Total	2035	15.03 (14.89–15.17)	–	4.34 (4.28–4.4)	–	52.87 (50.65–55.08)	–	2.82 (2.64–3)	–

**Table 2:** Variables per region

Regions	Total number of responses	Mean of 24-h on-call shifts in the last 3 months (CI 95%)	Mean of mandatory rest in the past 5 on-call shifts (CI 95%)	Percentage of a day-off after a Saturday 24-h on-call shift (CI 95%)	Mean working hours when not resting after a on-call shift (CI 95%)
Andalusia	414	15.31 (14.97–15.65)	4.45 (4.34–4.56)	49.63 (44.69–54.48)	3.03 (2.61–3.45)
Catalonia	27	12.41 (11.06–13.76)	4.44 (3.95–4.93)	50.00 (32.06–67.94)	1.19 (0.19–2.19)
Madrid	267	14.98 (14.64–15.32)	4.50 (4.36–4.64)	77.57 (1.95–82.36)	2.19 (1.74–2.64)
Valencia	205	15.38 (15.01–15.75)	4.54 (4.40–4.68)	10.55 (6.80–15.88)	1.92 (1.43–2.41)
Galicia	149	15.29 (14.89–15.69)	4.16 (3.91–4.41)	43.38 (35.00–52.15)	3.30 (2.70–3.90)
Castille and Leon	207	14.64 (14.11–15.17)	4.06 (3.85–4.27)	34.34 (28.04–41.25)	3.56 (2.93–4.18)
Basque Country	121	13.78 (13.20–14.36)	4.44 (4.19–4.69)	89.08 (81.71–93.82)	2.01 (1.34–2.68)
Canary Islands	65	14.43 (13.55–15.31)	4.77 (4.59–4.95)	87.69 (76.64–94.16)	2.11 (1.10–3.12)
Castille la Mancha	139	15.41 (14.90–15.92)	4.43 (4.24–4.62)	49.64 (41.03–58.26)	3.28 (2.37–4.19)
Murcia	111	15.12 (14.63–15.61)	4.29 (4.05–4.53)	70.91 (61.35–78.98)	3.40 (2.64–4.16)
Aragon	47	14.28 (13.24–15.32)	4.21 (3.76–4.66)	60.87 (45.39–74.54)	3.23 (1.87–4.59)
Balearic Islands	33	13.70 (12.58–14.82)	4.42 (3.95–4.89)	48.48 (31.17–66.15)	2.18 (0.52–3.84)
Extremadura	52	15.94 (15.38–16.50)	4.02 (3.58–4.46)	42.31 (29.01–56.74)	3.33 (2.10–4.56)
Asturias	46	16.83 (16.17–17.49)	3.93 (3.51–4.35)	65.22 (49.68–78.23)	2.67 (1.69–3.65)
Navarre	69	14.70 (13.73–15.67)	3.96 (3.55–4.37)	65.67 (52.97–76.56)	2.16 (1.36–2.96)
Cantabria	41	14.63 (13.14–16.12)	3.83 (3.29–4.37)	25.64 (14.60–42.43)	5.02 (3.27–6.77)
La Rioja	37	16.62 (16.16–17.08)	4.30 (3.85–4.75)	63.89 (46.21–78.66)	3.43 (2.19–4.67)
Ceuta & Melilla	5	15.60 (13.93–17.27)	4.60 (3.49–5.71)	100 (46.29–100)	5.6 (− 5.28–16.48)

Among the trainees surveyed, 80.49% of respondents exceeded the EWTD limit and had 5 or more on-call shifts per month. In 47.06% (8/17) of regions, the mean number of on-call shifts reported exceeded the limits imposed by the EWTD and national legislation.

The regions with the lowest mean of mandatory rest in the past 5 on-call shifts were Cantabria 3.83 (CI95% 3.29–4.37), Asturias 3.93 (CI95% 3.51–4.35), Navarre 3.96 (CI95% 3.55–4.37), Extremadura 4.02 (CI95% 3.58–4.46), and Castille and Leon 4.06 (CI95% 3.85–4.27), concentrating the highest resident physicians population who did not rest after 5 on-call shifts (Table 2); while by group of specialties Surgical-Medical 3.26 (CI95% 2.94–3.58), Surgical 3.26 (CI95% 3.01–3.51), and Diagnostic/Laboratory 4.41 (CI95% 4.08–4.74) were the ones who took the least rest (Table 1).

The percentage of a day-off after a Saturday on-call shift 51.60% (1050/2035). By group of specialties lowest rates were Surgical 22.64% (CI95% 17.31–28.98), Surgical-Medical 26.85% (CI95% 20.08–34.83), and Diagnostic/Laboratory 52.94% (CI95% 38.60–66.84) (Table 1). And by region, the lowest were Valencia 10.55 (CI95% 6.80–15.88), Cantabria 25.64 (CI95%14.60–42.43), and Castille and Leon 34.34 (CI95% 28.04–41.25) (Table 2).

The mean working hours when not resting after an on-call-shift were higher in the categories of: Primary Care 2.41 (CI95% 2.07–2.75), Surgical specialties 5.31 (CI95% 4.76–5.86), in females 2.73 (CI95% 1.33–3.09), and in the 2017 cohort of PGTs 3.62 (CI95% 2.76–4.48) (Table 1). And by region, the highest means were observed in Cantabria 5.02 (CI95% 3.27–6.77) and Castille and Leon 3.56 (CI95% 2.93–4.18). The lowest were seen in Catalonia 1.19 (CI95% 0.19–2.19), and Valencia 1.92 (CI95% 1.43–2.41) (Table 2).

The study found that the likelihood of having 12 or fewer 24-h on-call shifts in the past 3 months was significantly lower for Surgical specialties (OR 0.22 CI95% 0.12–0.39) and Surgical-Medical specialties (OR 0.48 CI95% 0.28–0.78) than for Primary Care. Additionally, the odds of having mandatory rest after on-call shifts were also significantly lower for Surgical specialties (OR 0.20 CI95% 0.14–0.27) and Surgical-Medical specialties (OR 0.28 CI95% 0.19–0.40) compared to Primary Care. Furthermore, the odds of having a day off after a Saturday 24-h on-call shift were significantly lower for Surgical specialties (OR 0.17 CI95% 0.11–0.23) and Surgical-Medical specialties (OR 0.21 CI95% 0.14–0.31) compared to Primary Care (Table 3).

**Table 3:** Data analysis by different variables

Variables	12 or less 24-h on-call shifts in the last 3 months (CI 95%)			Mandatory rest in the past 5 on-call shifts (CI 95%)			Percentage of a day-off after a Saturday 24-h on-call shift (CI 95%)			Not working after an on-call shift (CI95%)		
	%	OR	*p*	%	OR	*p*	%	OR	*p*	%	OR	*p*
*Type of specialties*												
Primary care	22.17 (19.10–25.57)	–-	–-	76.62 (73.17–79.76)	–	–	63.91 (60.08–67.58)	–	–	68.48 (64.76–71.97)	–	–
Other clinical	14.19 (12.06–16.63)	0.58 (0.45–0.75)	< 0.001	76.52 (73.65–79.17)	0.99 (0.79–1.26)	0.96	56.20 (52.92–59.42)	0.72 (0.59–0.89)	0.002	64.14 (60.96–67.20)	1.21 (0.98–1.50)	0.072
Surgical	5.96 (3.35–10.21)	0.22 (0.12–0.39)	< 0.001	39.45 (32.98–46.30)	0.20 (0.14–0.27)	< 0.001	22.64 (17.31–28.98)	0.17 (0.11–0.23)	< 0.001	21.56 (16.41–27.73)	7.90 (5.54–11.50)	< 0.001
Surgical-medical	11.95 (7.53–18.27)	0.48 (0.28–0.78)	0.005	47.80 (39.87–55.83)	0.28 (0.19–0.40)	< 0.001	26.85 (20.08–34.83)	0.21 (0.14–0.31)	< 0.001	32.08 (25.03–40.00)	4.60 (3.19–6.71)	< 0.001
Diagnostic/ laboratory	27.59 (17.05–41.11)	1.34 (0.73–2.45)	0.35	72.41 (8.89–82.95)	0.80 (0.45–1.50)	0.47	52.94 (38.60–66.84)	0.64 (0.36–1.13)	0.12	67.24 (53.54–78.65)	1.06 (0.59–1.85)	0.85
*Gender*												
Female	16.86 (14.93–18.97)	–-	–-	72 (69.43–74.36)	–	–	55.29 (52.54–58.01)	–	–	59.92 (57.23–62.56)	–	–
Male	14.61 (12.19–17.42)	0.84 (0.65–1.09)	0.190	66.95 (63.39–70.33)	0.79 (0.65–0.96)	0.018	48.24 (44.44–52.06)	0.75 (0.63–0.91)	0.003	56.03 (52.27–59.72)	1.17 (0.98–1.41)	0.090
*Cohort of PGT access*												
2017	11.76 (6.09–21.01)	0.57 (0.27–1.09)	0.11	58.82 (47.62–69.22)	0.56 (0.35–0.89)	0.014	39.76 (29.36–51.12)	0.58 (0.36–0.92)	0.022	42.35 (31.86–53.54)	2.02 (1.28–3.22)	0.003
2018	16.46 (12.63–21.11)	0.84 (0.58–1.21)	0.35	71.52 (66.14–76.36)	0.98 (0.72–1.33)	0.87	50.17 (44.39–55.94)	0.88 (0.67–2.27)	0.38	55.38 (49.71–60.92)	1.19 (0.91–1.58)	0.21
2019	15.77 (12.57–19.57)	0.80 (0.57–1.11)	0.19	66.67 (62.04–71)	0.78 (0.59–1.02)	0.065	50.80 (46.01–55.59)	0.90 (0.71–1.16)	0.43	55.18 (50.42–59.85)	1.20 (0.94–1.55)	0.14
2020	14.07 (11.45–17.16)	0.70 (0.51–0.95)	0.024	72.02 (68.22–75.53)	1.00 (0.78–1.29)	> 0.99	57.19 (53.08–61.2)	1.17 (0.93–1.48)	0.18	63.91 (59.92–67.72)	0.84 (0.66–1.06)	0.14
2021	18.94 (15.97–22.32)	–	–	72.01 (68.16–75.58)	–	–	53.30 (49.13–57.42)	–	–	59.73(55.62–63.71)	–	–

On the other hand, the odds of working after an on-call shift were significantly higher for Surgical specialties (OR 7.90 CI95% 5.54–11.59) and Surgical-Medical specialties (OR 4.60 CI95% 3.19–6.71) than for Primary Care. Moreover, the odds of working after an on-call shift were higher for the 2020 cohort (OR 0.84 CI95% 0.66–1.06) and significantly lower in the 2017 cohort (OR 2.02 CI95% 1.28–3.22) compared to the 2021 cohort (Table 3).

## Discussion and conclusion

This is the first study of its kind to examine the state-wide reality of duty hours and the compliance with European and national labor regulations of resident physicians in Spain. Previously it had only been studied with a regional scope in Madrid [6] and Valencia [5]. Our findings are congruent with those papers where the vast majority of residents exceed the maximum number of working hours per week set by the European Working Time Directive. However, there are significant geographical differences between regions, a situation which was not described in previous studies due to their regional lens. A similar trend to previous studies was observed when analyzing the underlying factors associated with non-compliance; namely the type of specialty (surgical specialties).

It is also the first European study of national reach which investigates EWTD compliance. Until now, most studies in Europe focused on a hospital level [10, 13] or a particular specialty [16]. There have been several discussions among specialties regarding the time limits imposed by the EWTD and training. In the case of anesthesia Waurick et al. [19] conclude that “[t]here are less measured clinical facts than political statements published”. In this sense, our results are more alarming than what had been hitherto described. In the only study focusing on gynecology trainees with a European scope, Goncalves-Henriques et al. [14] concluded that only 17.9% of the countries respected European working time regulations. However, they did not describe the extent of non-compliance in each country. Brohan et al. [3] analyze this problem in the specialty of Anesthesia in Ireland, in this case up to 70% of resident physicians exceeded the working time limits. In our study, this figure rises to 80% across all specialties.

The situation which has been described could potentially create a high-risk situation for the health and psychosocial well-being of resident physicians, hinder learning outcomes and could lead to suboptimal patient care. There is a vast body of literature on the physical and mental health consequences of sleep deprivation and work overload in doctors compared to the general population: increased motor vehicle accident risk [17], higher rates of depression, anxiety, burnout, drug and psychiatric medication abuse [7], to name only a few. Furthermore, both working in suboptimal conditions and the consequences it produces on the health of the residents could in turn pose a great risk to patient’s safety [17], when attended by these professionals who can spend up to 32 h working non-stop. In addition, some studies have explored the way in which excessive working hours could be detrimental to PGT outcomes insofar as learning could be significantly impaired [9, 12, 20].

The existing literature on the causes of this situation presents limited hypotheses for analysis. Only one study by Carrasco et al. [5] includes a qualitative section where residents provide their perspectives. Within this study, two main interconnected causes are emphasized: firstly, the hierarchical nature and the reliance on tradition in clinical cultures. The 24-h on-call shifts and the overall residency system have long been the established training system for specialists in Spain and Europe. Originally designed to ensure care continuity during periods of low demand, such as nights and weekends, this system has persisted. Secondly, the surge in demand and strain on healthcare systems in recent decades, exacerbated by economic crises and the COVID-19 pandemic. These two causes together create a work environment characterized by extreme conditions, with limited incentives for change due to the importance of the role of junior doctors in the medical workforce.

In terms of possible changes which could ameliorate these phenomena there are several implications both at policy and research levels. On the former, there is an urgent need for changes in national regulations to bring them into line with European regulations and for measures to be taken to ensure compliance. This would imply, in the first place, to change the regulatory framework (RD 1146/2006) and eliminate the mandate of working times based on on-call shifts, establishing it based on the number of worked hours. Secondly, to harmonize the different specialty training programs to the EWTD standards regarding the maximum weekly hours. And thirdly, to include in said law the weekly rest currently defined in the EWTD and ratified by the Spanish supreme court [18]. Furthermore, for surgical specialties, due to the importance of spending more time in operating room learning contexts, it would be interesting to review and extend training times in order to improve the acquisition of competencies as a possible reason for non-compliance with law. Lastly, in order to solve the situation, it would be of great interest to establish a time control system that ensures compliance with the maximum working time regulations.

On the latter, it is necessary to continue to carry out studies on the working conditions of health professionals. In this sense, it would be interesting to study in depth the causes of non-compliance with labor regulations and the consequences of exceeding the number of working hours, with both quantitative and qualitative studies, reflecting the personal experience of the resident and the power dynamics from which this phenomena might emanate in the healthcare field. In addition, it would be particularly useful to carry out studies which examine the association between non-compliance with weekly breaks, continuity of care and the number of hours worked by residents. Lastly, other overlapping areas which could benefit from further analysis are the deterioration of training quality, the toll on mental health of the professional and patient safety, as the existing studies have been carried out on other groups of healthcare professionals and not specifically on junior doctors working under the described conditions.

The present study has several limitations. Firstly, there may be a response bias as it is a voluntary survey, in which residents with less compliance with the corresponding breaks are more motivated to answer the survey. Secondly, the type of data collection method, since the data were collected through a self-administered online survey, there is limited control over the quality of the data. This method of data collection can lead to potential biases, lack of standardization, and response errors. Thirdly, the results regarding non-compliance with the EWTD could have been influenced by the effects of pandemic in the healthcare system. However, the studies which were conducted in Spain before the pandemic show a similar trend [5]. Finally, it was not possible to include too many resident physicians in Catalonia in the sample, since they use a system for managing their physicians' data which is independent of the centralized control of the Spanish Medical Organization making Catalan data underestimated.

In conclusion, the present study problematizes the working time of resident physicians and its compliance with working hours and rest periods. It is shown that the vast majority of residents exceed the maximum number of hours established by European regulations and a substantial percentage of residents do not take their daily rest after 24 h of uninterrupted work nor the established weekly rest periods. There are certain differences at the regional level and marked differences depending on the specialty, with surgical specialties standing out as those that least comply with the maximum work hours and rest periods established. All this is detrimental both to health and to the learning process, so measures are suggested at both the legislative and research levels.

### Supplementary Information

**Additional file 1. **Survey.

**Additional file 2. ** Medical residents by speciality, year of residency and percentage of responses.

**Additional file 3. ** Medical residents by region.

## Data Availability

All data generated or analyzed during this study are included in this published article.
