# Supplementary material for: Resident physician duty hours, resting times and European Working Time Directive compliance in Spain: a cross-sectional study
Source: Hum Resour Health. 2023 Aug 24;21:70. doi: 10.1186/s12960-023-00857-x (PMC10463816; doi:10.1186/s12960-023-00857-x)
Supplement: Supplementary file 1 — Additional file 1. Survey. [file 12960_2023_857_MOESM1_ESM.docx]

**Annex 1. SURVEY**

*This survey is only intended for resident physicians who are currently undergoing their postgraduate training and who elected placement from 2017. If you are not in this group please do not respond.*

**1 - Gender:**

( ) Male ( ) Female ( ) Other

**2 - In which year did you start your residency?**

( ) 2021 ( ) 2020 ( ) 2019 ( ) 2018 ( ) 2017

**3 - What is your specialty?**

Allergology

Pathological Anatomy

Anesthesiology and Resuscitation

Angiology and Vascular Surgery

Digestive system

Clinical Biochemistry

Cardiology

General and Digestive System Surgery

Oral and Maxillofacial Surgery

Orthopedic Surgery and Traumatology

Pediatric Surgery

Plastic Surgery

Thoracic Surgery

Dermatology

Endocrinology and Nutrition

Clinical Pharmacology

Geriatrics

Hematology and Hemotherapy

Immunology

Occupational Medicine

Family and Community Medicine

Physical Medicine and Rehabilitation

Intensive Care Medicine

Internal Medicine

Nuclear Medicine

Preventive Medicine and Public Health

Microbiology and Parasitology

Nephrology

Pneumology

Neurosurgery

Clinical Neurophysiology

Neurology

Obstetrics and Gynecology

Ophthalmology

Medical Oncology

Radiation Oncology

Otorhinolaryngology

Pediatrics

Psychiatry

Radiodiagnostics

Rheumatology

Urology

**4 - What is your Community / Autonomous City of practice?**

Andalusia

Aragon

Principality of Asturias

Balearic Islands

Canary Islands

Cantabria

Castille la Mancha

Castille and Leon

Catalonia

Ceuta

Valencian Community

Extremadura

Galicia

La Rioja

Community of Madrid

Melilla

Region of Murcia

Navarre

Basque Country

**Throughout this survey, the on-call concept will be considered as:**

*-Working hours which are done in-house with shifts of 17 and 24 hours with physical presence which do not correspond to the ordinary working schedule of 37,5 hours per week.*

*-Mandatory rest: the obligatory rest after a 24h on-call shift.*

**5 - In the last month, how many on-call shifts have you done?**

( ) 0 ( ) 1 ( ) 2 ( ) 3 ( ) 4 ( ) 5 ( ) 6 ( ) 7 ( ) 8 ( ) 9 ( ) 10

**- Which month have you taken as a reference?**

( ) November 2021 ( ) December 2021 ( ) January 2022 ( ) February 2022 ( ) March 2022 ( ) March 2022

**- In the last three months, how many on-call shifts have you done in total? (ELIMINATING QUESTION if 0)**

( ) 0 ( ) 1 ( ) 2 ( ) 3 ( ) 4 ( ) 5 ( ) 6 ( ) 7 ( ) 8 ( ) 9 ( ) 10 .... ( ) 30

**- In how many of the last five on-call shifts you were able to take the mandatory rest period (did you have time off the following day)?**

( ) 0 ( ) 1 ( ) 2 ( ) 3 ( ) 4 ( ) 5

**- After your last Saturday on-call shift, were you able to rest on the following Monday or Friday (minimum weekly rest period of 36 hours or compensatory rest of 48 hours in 15 days)?**

( ) Not applicable ( ) Yes ( ) No ( ) No

**- In the last on-call shift that you did not rest afterwards, how many hours did you work after the end of that on-call shift?**

( ) I have rested on all on-call shift ( ) 1 ( ) 2 ( ) 3 ( ) 4 ( ) 5 ( ) 6 ( ) 7 ( ) 8 ( ) 9 ( ) 10 ( ) 10 .... ( ) 24

**- Does the training unit where you are on call use a system of substitution lists or incidents? (Systems in which when a resident is absent, another resident is obliged to cover his/her position; including situations with a notice period of 24h or less)**

( ) Yes ( ) No
