# Supplementary material for: Resident physician duty hours, resting times and European Working Time Directive compliance in Spain: a cross-sectional study
Source: Hum Resour Health. 2023 Aug 24;21:70. doi: 10.1186/s12960-023-00857-x (PMC10463816; doi:10.1186/s12960-023-00857-x)
Supplement: Supplementary file 2 — Additional file 2. Medical residents by speciality, year of residency and percentage of responses. [file 12960_2023_857_MOESM2_ESM.docx]

Annex 2: Medical residents by speciality, year of residency and percentage of responses.

| **Specialty** | **Type of specialty** | **1st year** | **2nd year** | **3rd year** | **4th year** | **5th year** | **Total** | **N of responses** | **Percentage** |
| --- | --- | --- | --- | --- | --- | --- | --- | --- | --- |
| Allergology | Other Clinical | 72 | 67 | 61 | 54 | 0 | 254 | 20 | 7.9% |
| Clinical Analysis | Diagnostic/ Laboratory | 26 | 15 | 19 | 18 | 0 | 78 | 2 | 2.6% |
| Pathological anatomy | Diagnostic/ Laboratory | 122 | 117 | 104 | 97 | 0 | 440 | 4 | 0.9% |
| Anesthesiology and resuscitation | Other Clinical | 393 | 387 | 344 | 329 | 0 | 1453 | 83 | 5.7% |
| Vascular angiology and surgery | Surgical | 46 | 38 | 35 | 36 | 35 | 190 | 13 | 6.8% |
| Digestive system | Other Clinical | 178 | 172 | 161 | 156 | 0 | 667 | 49 | 7.3% |
| Clinical Biochemistry | Diagnostic/ Laboratory | 4 | 4 | 6 | 4 | 0 | 18 | 0 | 0.0% |
| Cardiology | Other Clinical | 181 | 180 | 168 | 166 | 159 | 854 | 66 | 7.7% |
| Cardiovascular surgery | Surgical | 21 | 25 | 24 | 20 | 25 | 115 | 4 | 3.5% |
| General and digestive surgery | Surgical | 227 | 212 | 198 | 189 | 187 | 1013 | 76 | 7.5% |
| Oral and maxillofacial surgery | Surgical | 32 | 32 | 32 | 31 | 31 | 158 | 13 | 8.2% |
| Orthopedic surgery and traumatology | Surgical | 279 | 267 | 243 | 242 | 235 | 1266 | 78 | 6.2% |
| Pediatric surgery | Surgical | 20 | 22 | 23 | 20 | 22 | 107 | 9 | 8.4% |
| Aesthetic and repair plastic surgery | Surgical | 51 | 41 | 40 | 37 | 38 | 207 | 8 | 3.9% |
| Thoracic surgery | Surgical | 23 | 17 | 26 | 17 | 19 | 102 | 2 | 2.0% |
| Medical-Surgical Dermatology | Surgical-Medical | 112 | 108 | 94 | 88 | 0 | 402 | 18 | 4.5% |
| Endocrinology and nutrition | Other Clinical | 89 | 87 | 81 | 78 | 0 | 335 | 27 | 8.1% |
| Clinical Pharmacology | Diagnostic/ Laboratory | 19 | 18 | 16 | 16 | 0 | 69 | 4 | 5.8% |
| Geriatrics | Other Clinical | 95 | 78 | 66 | 65 | 0 | 304 | 25 | 8.2% |
| Hematology and hemotherapy | Other Clinical | 138 | 133 | 122 | 124 | 0 | 517 | 38 | 7.4% |
| Immunology | Diagnostic/ Laboratory | 17 | 12 | 10 | 11 | 0 | 50 | 2 | 4.0% |
| Occupational Medicine | Other Clinical | 102 | 96 | 65 | 63 | 0 | 326 | 23 | 7.1% |
| Family and community medicine | Primary Care | 2338 | 2224 | 1912 | 1810 | 0 | 8284 | 663 | 8.0% |
| Physical Medicine and Rehabilitation | Other Clinical | 123 | 117 | 99 | 100 | 0 | 439 | 24 | 5.5% |
| Intensive medicine | Other Clinical | 186 | 178 | 163 | 162 | 159 | 848 | 59 | 7.0% |
| Internal Medicine | Other Clinical | 389 | 360 | 334 | 321 | 308 | 1712 | 142 | 8.3% |
| Nuclear medicine | Diagnostic/ Laboratory | 43 | 47 | 45 | 42 | 0 | 177 | 3 | 1.7% |
| Preventive medicine and public health | Other Clinical | 107 | 105 | 69 | 65 | 0 | 346 | 8 | 2.3% |
| Microbiology and parasitology | Diagnostic/ Laboratory | 44 | 38 | 30 | 20 | 0 | 132 | 7 | 5.3% |
| Nephrology | Other Clinical | 106 | 104 | 97 | 92 | 0 | 399 | 34 | 8.5% |
| Pneumology | Other Clinical | 135 | 126 | 115 | 110 | 0 | 486 | 33 | 6.8% |
| Neurosurgery | Surgical | 41 | 45 | 45 | 41 | 39 | 211 | 15 | 7.1% |
| Clinical Neurophysiology | Other Clinical | 54 | 44 | 40 | 42 | 0 | 180 | 1 | 0.6% |
| Neurology | Other Clinical | 142 | 138 | 129 | 123 | 0 | 532 | 33 | 6.2% |
| Obstetrics and Gynecology | Surgical-Medical | 275 | 271 | 266 | 255 | 0 | 1067 | 68 | 6.4% |
| Ophthalmology | Surgical-Medical | 202 | 195 | 176 | 171 | 0 | 744 | 25 | 3.4% |
| Medical oncology | Other Clinical | 144 | 138 | 120 | 120 | 114 | 636 | 65 | 10.2% |
| Radiation oncology | Other Clinical | 69 | 64 | 62 | 57 | 0 | 252 | 11 | 4.4% |
| Otorhinolaryngology | Surgical-Medical | 100 | 99 | 83 | 79 | 0 | 361 | 22 | 6.1% |
| Pediatrics and specific areas | Other Clinical | 491 | 481 | 433 | 423 | 0 | 1828 | 126 | 6.9% |
| Psychiatry | Other Clinical | 288 | 272 | 248 | 241 | 0 | 1049 | 56 | 5.3% |
| Radiodiagnosis | Diagnostic/ Laboratory | 260 | 257 | 229 | 223 | 0 | 969 | 36 | 3.7% |
| Rheumatology | Other Clinical | 75 | 64 | 56 | 54 | 0 | 249 | 14 | 5.6% |
| Urology | Surgical-Medical | 128 | 120 | 105 | 101 | 97 | 551 | 26 | 4.7% |
| National Total | National Total | 7987 | 7615 | 6794 | 6513 | 1468 | 30377 | 2035 | 6.7% |
| N of responses | National Total | 586 | 604 | 444 | 316 | 85 | 2035 |  |  |
| Percentage | National Total | 7.3% | 7.9% | 6.5% | 4.9% | 5.8% | 6.7% |  |  |
