# Supplementary material for: Resident physician duty hours, resting times and European Working Time Directive compliance in Spain: a cross-sectional study
Source: Hum Resour Health. 2023 Aug 24;21:70. doi: 10.1186/s12960-023-00857-x (PMC10463816; doi:10.1186/s12960-023-00857-x)
Supplement: Supplementary file 3 — Additional file 3. Medical residents by region. [file 12960_2023_857_MOESM3_ESM.docx]

Annex 3: Medical residents by region.

| **Region** | **N Medical Residents** | **N of responses** | **Percentage** |
| --- | --- | --- | --- |
| Andalusia | 4890 | 414 | 8.47% |
| Aragon | 951 | 47 | 4.94% |
| Asturias | 668 | 46 | 6.89% |
| Balearic Islands | 591 | 33 | 5.58% |
| Canary Islands | 1174 | 65 | 5.54% |
| Cantabria | 479 | 41 | 8.56% |
| Castilla–La Mancha | 1235 | 139 | 11.26% |
| Castile and León | 1708 | 207 | 12.12% |
| Catalonia | 4707 | 27 | 0.57% |
| Valencia | 2949 | 205 | 6.95% |
| Extremadura | 742 | 52 | 7.01% |
| Galicia | 1485 | 149 | 10.03% |
| Ceuta and Melilla | 35 | 5 | 14.29% |
| La Rioja | 184 | 37 | 20.11% |
| Madrid | 5546 | 267 | 4.81% |
| Murcia | 987 | 111 | 11.25% |
| Navarre | 571 | 69 | 12.08% |
| Basque Country | 1475 | 121 | 8.20% |
| National Total | 30377 | 2035 | 6.70% |
